# *Dual Specificity Phosphatase* (*DUSP22*) promoter hypomethylation in cell-free DNA is associated with rheumatoid arthritis and its radiographic severity

**DOI:** 10.3389/fmed.2026.1730527

**Published:** 2026-02-03

**Authors:** Lissette Delgado-Cruzata, Milena Rodriguez Alvarez, Alorah D. Bliese, Toni-Ann T. Bravo, Nicholas Petraco, Edgardo Guzman, Wallis Tavarez, Menachem Gold, Shante Hinson

**Affiliations:** 1 John Jay College of Criminal Justice, City University of New York, New York, NY, United States; 2 State University of New York Downstate Medical Center, Brooklyn, NY, United States; 3 Boehringer Ingelheim Pharmaceuticals, Ridgefield, CT, United States; 4 New York City Health + Hospitals, Lincoln Medical Center, Bronx, NY, United States

**Keywords:** cell free-DNA, DNA methylation, *DUSP22*, epigenetics, Hispanic, rheumatoid arthritis

## Abstract

**Objectives:**

While several advances have been made in the last decade, reliable biomarkers for disease activity, prognosis, and response to treatment of rheumatoid arthritis (RA) have yet to be identified. In previous studies, *DUSP22* DNA methylation changes were found to be associated with RA and erosive disease. We conducted a pilot study to investigate plasma cell-free DNA (cfDNA) methylation in *DUSP22* in a cohort of RA patients and healthy controls. We also investigate *DUSP22* DNA methylation associations with RA clinical characteristics and treatment.

**Methods:**

DNA was isolated from plasma from 27 RA patients who satisfied the ACR criteria, and 18 healthy controls. *DUSP22* DNA methylation was determined by pyrosequencing. Statistical analysis identified group differences and associations with RA clinical measures.

**Results:**

RA patients had lower cfDNA *DUSP22* DNA methylation at one specific DNA methylation site in the promoter region when compared to controls (36.7 ± 3.3% for RA versus 46.9 ± 2.6% for controls, age-adjusted-*p* = 0.049). For RA patients, age was associated with a significant decrease in *DUSP22* DNA methylation for all sites and the promoter region (β_*mean*_ = −0.64, *p* = 0.02). Lower DNA methylation was also associated with increased joint space narrowing (ρ_*CpGMean*_ = −0.40, *p* = 0.05).

**Conclusion:**

Our pilot study is the first to evaluate cfDNA methylation association to RA clinical characteristics. These exploratory findings suggest that *DUSP22* cfDNA methylation may represent a promising non-invasive biomarker in rheumatoid arthritis, a hypothesis that warrants validation in larger and ethnically diverse populations.

## 1 Introduction

Rheumatoid arthritis (RA) is a chronic autoimmune disease that causes persistent inflammation, pain, and destruction of the joints (1). The etiology of RA is complex due to the combined effect of genetic and environmental factors (2). Thus, epigenetics has emerged as a key integrative mechanism, supported by epigenetic modifications observed in RA patients (3–10). DNA methylation, the most studied epigenetic modification, is the addition of a methyl group to the fifth carbon of cytosine nucleotides when next to guanines in the DNA, called CpG sites (11). This modification can regulate gene expression by limiting access to the transcription machinery to the promoters of genes. Disease-associated DNA methylation patterns have been identified in a variety of cell types from RA patients, including peripheral blood mononuclear cells (PBMCs), B-cells, T-cells, and fibroblast-like synoviocytes (FLS) (4–10). Several differentially methylated genes have been identified in the synovium of individuals with RA, which encode proteins involved in inflammatory response (5, 12, 13). Other research has found B- and T-lymphocyte DNA methylation of specific genes to be associated with established RA, early RA (RA in naïve patients) and response to disease-modifying antirheumatic drugs (DMARDs) (4, 5, 7). Combined, these studies have shown that DNA methylation plays an important role in RA and potentially contributes to the persistent inflammation these patients experience.

Previous research identified DNA methylation changes in the *Dual Specificity Phosphatase 22* (*DUSP22*) gene promoter to be associated with RA (4, 9). *DUSP22*, also known as *JKAP*, encodes a phosphatase belonging to a family of enzymes that can dephosphorylate serine/threonine and tyrosine residues (14). *DUSP22* enzyme has an immuno-modulator role; this is evidenced by its regulation of c-Jun N-terminal kinases (JNK), signal transducer activator of transcription-3 (STAT3), and the lymphocyte-specific protein tyrosine kinase (LCK) (15–17). Findings show that *DUSP22* can activate JNK, one of the mammalian mitogen-activated protein kinases (MAPKs) and does this independently of its phosphatase activity. This activation can occur as a response to stress, growth, and apoptosis (15, 18). In addition, *DUSP22* can dephosphorylate STAT3 and prevent it from translocating into the nucleus to promote the transcription of pro-inflammatory cytokines such as IL6 (16). Finally, data suggests that *DUSP22* inactivates LCK in the T-cell receptor (TCR) signaling pathway leading to autoimmunity and inflammation (17). All these mechanisms have been identified as important in the etiology and progression of RA, making *DUSP22* an important molecular target in this disease.

While most studies investigating epigenetic biomarkers of RA have focused on DNA extracted from lymphocytes and FLS, there is evidence that cell free-DNA (cfDNA) plays an important role in autoimmune diseases (19–22). Data has shown that there is more cfDNA in the plasma of RA patients than in controls (23–25). Studies have also found that better clinical outcomes are observed in individuals with RA in which the amounts of cfDNA increase after DMARDs therapy and that the use of cfDNA amounts along with a measure of anti-citrullinated peptide antibody (ACPA) might be a better diagnostic tool in RA than using ACPA alone (22, 25). However, studies have not investigated how cfDNA methylation markers relate to RA clinical characteristics. This new area of research could improve our understanding of RA pathogenesis. Assessing cfDNA methylation offers a minimally invasive approach that could complement serologic biomarkers in RA and facilitate disease monitoring. Here, we propose to investigate DNA methylation biomarkers in the Epigenetics of Rheumatoid Arthritis (ERA) study. We researched whether DNA methylation biomarkers can be measured in plasma cfDNA, and whether levels of DNA methylation in *DUSP22* differ between Hispanic RA and healthy individuals. We also explored their associations with radiographic findings and disease activity measures in this population.

## 2 Materials and methods

### 2.1 Study participants

This was a small, proof-of-concept study designed to investigate at *DUSP22* promoter DNA methylation levels in plasma cfDNA from patients with RA and to explore possible clinical associations in a mainly Hispanic group. The number of participants was limited by the availability of well-characterized cases and controls who met strict inclusion criteria but was considered suitable for an exploratory study. Twenty-seven RA patients, who meet the 2010 ACR classification criteria for RA (26), and eighteen healthy individuals, all 18 years or older, were recruited to the Epigenetics of Rheumatoid Arthritis (ERA) study. Recruitment took place from May 2016 to March 2018. The RA cohort attended the Rheumatology clinic of New York City Health + Hospitals/ Lincoln Hospital, and healthy individuals learned about the study via open advertisement. Once a patient agreed to participate in the study and provided consent, their medical records were reviewed to assess inclusion/exclusion criteria. RA patients with any of the following conditions were excluded: other autoimmune diseases, type 2 diabetes mellitus, entrapment neuropathies, radiculopathies, recent sepsis (period less than 6 months), chronic infections (Hepatitis C/B, HIV), previous and current intravenous drug use, chronic kidney disease (CKD 2), chronic heart failure, liver cirrhosis, patients taking more than 20 mg daily of corticosteroids at the time of recruitment, cancer, recent organ transplant, pregnancy, recent significant trauma (less than 6 months including admission to the hospital, fractures), recent major surgery (all recent surgeries within 4 months, except: cataracts, ambulatory or superficial procedures, breast lumpectomy, colonoscopy, and/or endoscopy). Healthy controls were screened by questionnaire and interview to exclude a past medical history of autoimmune disease, chronic inflammatory or systemic conditions, malignancy, or chronic infection, and were free of symptoms of acute illness at the time of sample collection. Informed consent was obtained from all subjects involved in the study. All participants completed a questionnaire including demographic data and in the case of RA patients, symptoms and clinical measures associated with RA were extracted from their patient records. Peripheral whole blood samples were collected in green top. Heparin containing tubes with randomly assigned IDs, and transported to the laboratory at John Jay College, College of the University of New York (CUNY) in sealed containers. The study was conducted according to the guidelines of the Declaration of Helsinki and approved by the Institutional Review Board of New York City Health + Hospitals/ Lincoln Hospital in New York.

### 2.2 Clinical measures

In this study, we define seropositive disease by the presence of either ACPA or rheumatoid factor (RF). Conversely, seronegative status meant neither factor was positive. RA patients were assessed utilizing the following clinical measures: Clinical Disease Activity Index (CDAI), Simple Erosive Narrowing Score (SENS), CDAI considers the number of swollen and tender joints as well as the patient and provider assessments of global disease activity to obtain a score ranging from 0.0 to 76.0, with disease activity increasing with higher values (27). SENS is a scoring system ranging from 0 to 86 that determines the presence and number of joint erosions (32 for hands and 12 for feet) and the presence and number of joint space narrowing (30 for the hands and 12 for the feet) (28, 29).

### 2.3 DNA extraction and methylation analysis of *DUSP22*

**FIGURE 1:**
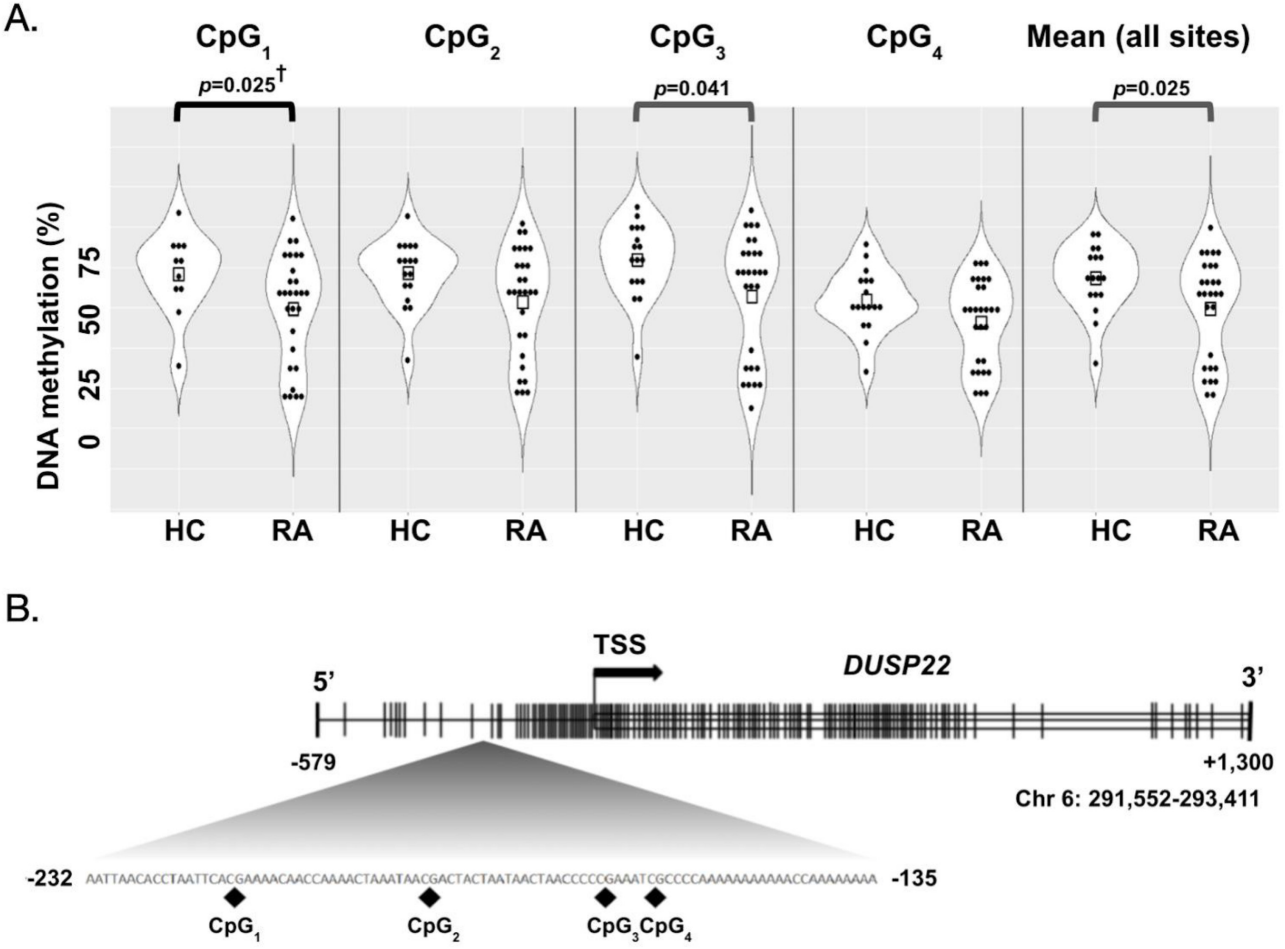
*DUSP22* DNA methylation in the Epigenetics of Rheumatoid Arthritis (ERA) study population. **(A)** Violin plots showing percent DNA methylation at each CpG site and in average for the *DUSP22* promoter region for healthy individuals (HC) and RA patients (RA). Measurements for twenty-six (26) RA patients, and eighteen (18) HC were included in this comparison. Unadjusted Mann-Whitney *p* ≤ 0.05 are included above the brackets, † is used to indicate the age-adjusted *p* ≤ 0.05. **(B)** Map of the *DUSP22* DNA methylation region measured in this study. A 1,859 kb fragment in chromosome 6 that includes the start of the *DUSP22* gene is shown. The transcription start-site (TSS) of the gene and 579 bp of its promoter region are both indicated. Across the sequence, lines are used to indicate the presence of CpG sites. The sequence to analyze from the pyrosequencing assay, located between –232 bp and –135 bp upstream of the *DUSP22* promoter, is included in the insert (4). The four CpG sites measured in this study are indicated in this sequence with the nomenclature used throughout the study. All genomic locations correspond to genome assembly 37.

Plasma was separated from whole blood by centrifugation. DNA was extracted from 1 mL of plasma using an UltraSens Virus Kit (Qiagen) according to the manufacturer’s instructions. We obtained typical yields for extractions from plasma ranging from 5 to 15 ng/mL plasma. From the extracted DNA, 2.5–5 μL were used for bisulfite conversion using the EZ DNA Methylation-Gold kit (Zymo Research) following the kit’s protocol. Bisulfite converted DNA was eluted in 15 μl of water and 2.5–5 μl were used for Pyromark PCR amplification (Qiagen). The assay targets a 177-bp DNA fragment in the *DUSP22* promoter region (4). Figure 1 shows the location of all CpG sites of the *DUSP22* promoter measured in this study and previously reported to be associated with RA in previous studies (Figures 1B and Supplementary Figure 1) (4, 9). The forward primer 5′-GGTAGGGGGTTTTTAGATTTTTT-3′ was biotinylated in the 5′ end, and the sequence of the reverse primer was 5′-CCCCCAACCTAAATCTACC-3′. The annealing temperature used in amplification reactions was 56 °C and 38 cycles. Pyrosequencing was carried out in a PyroMark Q24 (Qiagen) following the manufacturer’s instructions using 5′-CCCAAAAACCAAACCTCT-3′ as the sequencing primer and the following sequence to analyze 5′-AATTAACACCTAATTCACRAAAACAACCAAAACTAAATAAC RACTACTAATAACTAACCCCCRAAATCRCCCCAAAAAAAAA AACCAAAAAAAA-3′. Pyrosequencing measured DNA methylation levels of four CpG sites (Figure 1B). DNA methylation analysis was performed using the PyroMark Q24 Advanced 3.0.0 software. DNA methylation status was reported as the average percent methylation for the four CpG sites, or by individual CpG sites as indicated. Each amplification and pyrosequencing run included fully methylated and unmethylated DNA standards (Zymo Research) as controls. No-template controls were also included in all runs. Technical staff carrying out the DNA methylation analysis were blinded by RA-status or any demographic characteristic associated with the study participant as all samples were de-identified and assigned a random ID after collection. Coefficient of variation for the assay was 6.50%.

### 2.4 Statistical analysis

We used statistical tools to compare demographic characteristics, and to explore the associations of the percent of DNA methylation of *DUSP22* in RA patients and controls and with clinical variables. Mean values and standard deviation (SD) are reported for all measures including DNA methylation percentages, clinical and demographic variables. Normality of data distribution was assessed for each CpG site using the Shapiro–Wilk test, and equality of variances across groups was verified by Levene’s test. We used a *t*-test to compare demographic characteristics and a non-parametric Mann-Whitney test to compare DNA methylation levels at each site and across the region between RA patients and controls. In addition, we carried out linear regression modeling to examine the relationship between DNA methylation levels in both RA patients and healthy controls as a function of age. We logit-transformed DNA methylation percentages and carried out ANCOVA to compare DNA methylation levels between RA patients and controls while adjusting for age. Age was included as a covariate in these analyses, as it significantly differed between RA patients and the controls and could confound the association with DNA methylation levels. Spearman tests were conducted to determine the correlations between DNA methylation levels and clinical measures. In this analysis, to adjust for age, residuals from separate linear regressions of each variable on age were computed and correlated. For interpretation, ANCOVA effect sizes were expressed as partial eta-squared (η^2^). Values were interpreted according to Cohen’s thresholds, with 0.01 considered small, 0.06 medium, and 0.14 large. Statistical significance was defined as *p* ≤ 0.05, while results with *p*-values between 0.05 and 0.10 were described as trends. SPSS v-31 was used for analysis.

### 2.5 Data availability statement

The datasets generated for this study will be provided to interested researchers upon request.

## 3 Results

The demographic and clinical characteristics of the ERA study participants are shown in Table 1. Participants were mostly females and of Hispanic ethnicity (Table 1). Gender and race/ethnicity were similar between RA patients and controls; however, RA patients were significantly older than the controls (Table 1). Only three individuals in this sample were smokers; therefore, we did not include that variable in our analysis. Treatment data was available for twenty-six RA patients; 93.2% of them were on DMARDs alone or in combination with biologics (Table 1). The most common DMARDs used was hydroxychloroquine (61.5%), followed by methotrexate (53.8%). A total of 38.5% of participants were under combined hydroxychloroquine and methotrexate treatment. The most common biologic used was *TNF*α inhibitors and it was used by 23.1% of patients. In Figure 1, the percentages of DNA methylation for all CpG sites and the mean in the *DUSP22* promoter region measured in this study are presented (Figure 1A). The mean percentage of *DUSP22* DNA methylation in the promoter region was significantly lower in RA patients (36.5 ± 3.1%) compared with healthy controls (47.1 ± 2.4%, *p* = 0.025; Figure 1A). At the single-site level, CpG1 and CpG3 showed significantly lower methylation in RA patients (CpG1: 36.7 ± 3.3% and CpG3: 40.4 ± 3.7%) versus for healthy individuals (CpG1: 46.9 ± 2.6%, *p* = 0.025; CpG3: 52.7 ± 2.7%, *p* = 0.041), while CpG2 and CpG4 showed no significant differences. After adjusting for age, comparison of DNA methylation between RA patients and controls at CpG1 remained statistically significant (*p* = 0.049) with an effect size in the large range (η^2^ = 0.140), indicating a substantial difference despite the limited sample size (Figure 1A and Supplementary Table 1).

**TABLE 1:** Demographic and clinical characteristics of Epigenetics of Rheumatoid Arthritis (ERA) study participants.

Characteristics	Controls	RA
No. of subjects	18	27
Age, mean years ± SD	42.1 ± 14.1	57.8 ± 11.4
Female, %	66.7%	77.8%
Hispanic, %	88.9%	88.9%
African American, %	5.5%	11.1%
Other races, %	5.5%	0.0%
Positive for ACPA, %	–	69.2%
Positive for RF, %	–	61.5%
Positive for ACPA and RF, %	–	53.8%
CDAI	–	16.1 ± 2.0
SENS (0 = no erosions/joint space narrowing, 172 = erosions and joint narrowing)	–	20.4 ± 3.9
Mean of erosions number	–	4.6 ± 1.9
Mean of joint narrowing spaces	–	16.7 ± 2.8
DMARDs only (%)	–	34.8%
Biologics only (%)	–	8.7%
Biologics and DMARDs, %	–	56.5%

**FIGURE 2:**
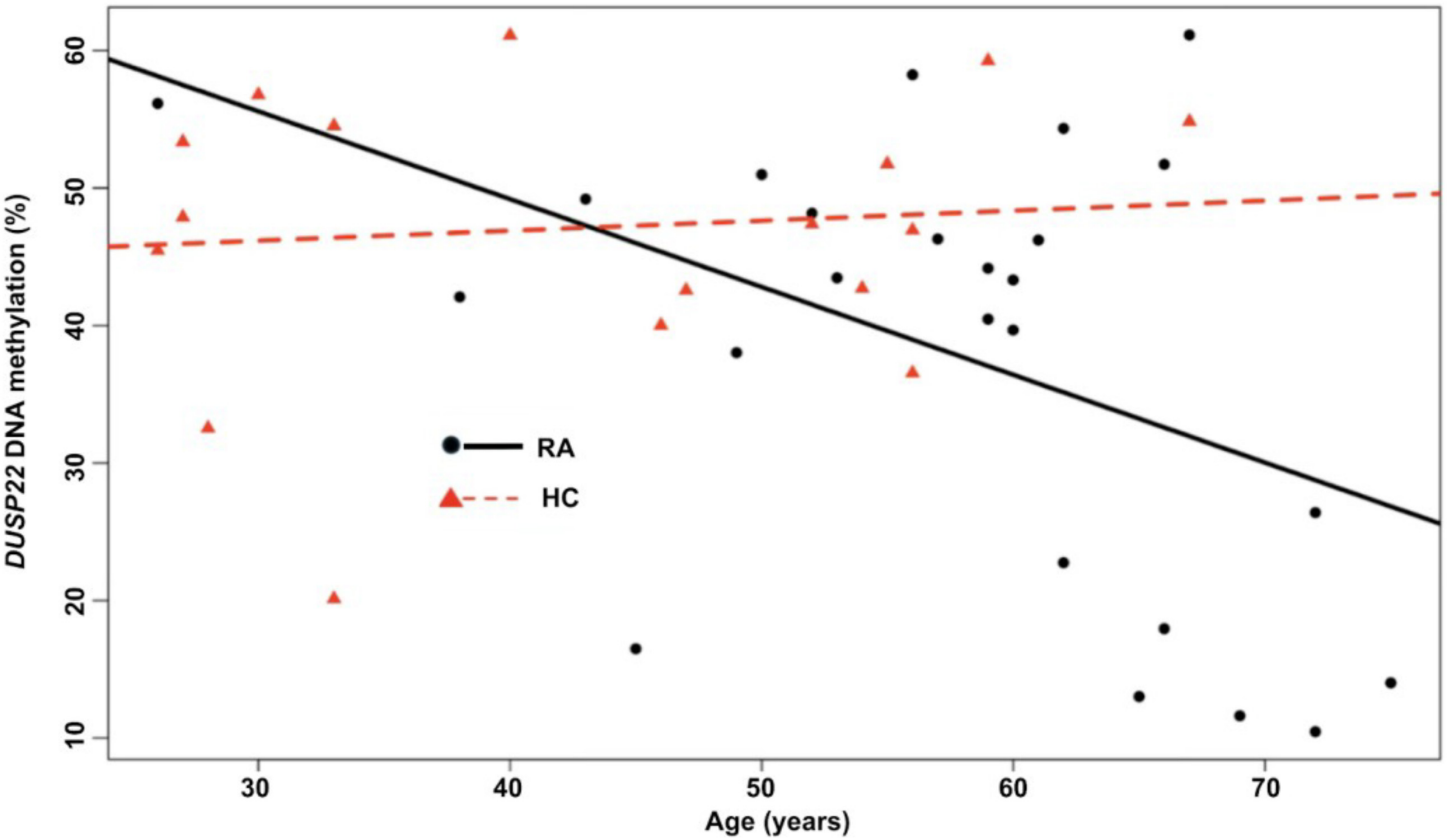
*DUSP22* DNA methylation and age in the ERA study population. Raw mean DNA methylation of the *DUSP22* promoter region are presented as black circles for RA patients (RA) and red triangles for healthy controls (HC).

Because age differed between RA patients and healthy controls, we used linear regression to examine the association between age and *DUSP22* DNA methylation in each group. Among RA patients, age was significantly associated with lower DNA methylation at all CpG sites. (β_*CpG1*_ = −0.64, *p* = 0.03; β_*CpG2*_ = −0.68, *p* = 0.02; β_*CpG3*_ = −0.73, *p* = 0.03 and β_*CpG4*_ = −0.51, *p* = 0.03) and for the mean promoter DNA methylation (β_*mean*_ = −0.64, *p* = 0.02; Figure 2). No association with age was observed in controls (β_*mean*_ = 0.07, *p* = 0.69; Figure 2).

We next examined the correlations between *DUSP22* methylation and clinical measures, including disease activity and radiographic findings. In RA patients, lower *DUSP2*2 methylation correlated with greater radiographic damage severity. Results are shown in Table 2. A significant negative correlation was observed between SENS and CpG2 methylation (ρ_*CpG2*_ = −0.40, *p* = 0.05), with similar trends for CpG1 (ρ_*CpG1*_ = −0.31, *p* = 0.07), CpG3 (ρ_*CpG3*_ = −0.34, *p* = 0.09), and the mean promoter DNA methylation (ρ_*mean*_ = −0.37, *p* = 0.09). Lower DNA methylation at CpG1, CpG3, and the mean promoter DNA methylation also correlated with greater joint space narrowing (ρ_*CpG1*_ = −0.41, *p* = 0.04; ρ_*CpG3*_ = −0.43, *p* = 0.03, and ρ_*mean*_ = −0.40, *p* = 0.05, respectively), and these associations remained consistent after adjusting for age. No significant correlations were observed with CDAI. We also found no differences by medication use in *DUSP22* DNA methylation levels at specific sites and with the mean promoter (Supplementary Table 2).

**TABLE 2:** Spearman correlation coefficients of the associations of clinical characteristics and DNA methylation at the *DUSP22* promoter in the rheumatoid arthritis (RA) patients of the Epigenetics of Rheumatoid Arthritis (ERA) population.

Clinical characteristics	% DNA methylation				
	CpG 1	CpG 2	CpG 3	CpG 4	Mean
SENS	−0.37	−**0.40**	−0.34	−0.31	−0.36
*P*-value	0.07	**0.05**	0.09	0.13	0.07
Joint narrowing	**−0.41**	−**0.43**	−0.37	−0.35	**−0.40**
*P*-value	**0.04**	**0.03**	0.07	0.09	**0.05**

*P*-values ≤ 0.05 have been bolded to highlight significant correlations.

## 4 Discussion

In this pilot study, we investigated DNA methylation of the *DUSP22* promoter in the plasma cfDNA of predominantly Hispanic RA patients and healthy controls. We found that DNA methylation at the *DUSP22* promoter was lower in individuals with RA than controls, for RA patients we also observed a trend of lower *DUSP22* DNA methylation with increasing age. We also found that DNA methylation at specific sites and overall, in the region was associated with radiological measures of disease (erosive disease and joint narrowing). These associations suggest that lower levels of *DUSP22* methylation might be a feature of the disease and that plasma cfDNA has the potential to be a relevant biomarker in RA, if these findings are replicated in larger cohorts. A previous study identified T-lymphocyte DNA hypermethylation of the *DUSP22* promoter in RA (4). Similarly, studies performed on other autoimmune disorders support RA findings in T-lymphocytes. Sjögren syndrome patients were found to have higher levels of *DUSP22* DNA methylation in T-lymphocytes DNA when compared to controls, and a study carried out in systemic lupus erythematosus (SLE) patients found them to have less *DUSP22* T-lymphocyte expression as well as a negative association between SLE disease activity and *DUSP22* expression (30, 31). Less *DUSP22* expression could be the result of epigenetic regulation of gene expression, in which higher *DUSP22* DNA methylation would lead to lower expression of this gene. One important distinction between our study and those carried out before is that DNA methylation here was measured in plasma cfDNA while these previous studies measured this epigenetic biomarker in T-lymphocytes. DNA methylation is a tissue specific molecular mark, but cfDNA does not represent one unique cell type or tissue. While knowledge about which proportions of DNA end up in the cfDNA it is not fully known, and how these proportions might change considering demographic and clinical characteristics, some studies have shed clarity on this issue. In one study that measured mitochondrial DNA in plasma and synovial fluid, only RA patients with substantial amounts of mitochondrial DNA in the synovial fluid had equally large amounts of plasma mitochondrial DNA (23). Another study found that the DNA methylation pattern in synovial fluid from RA patients is like the pattern in synovial cells and has suggested that RA cfDNA originated mainly in the synovium (32). Interestingly, exposure to IL-1 leads to lower DNA methylation in fibroblast-like synoviocytes, and studies in SLE patients have shown their DNA is mostly hypomethylated (33, 34). Those findings suggest that synovial DNA might be part of the cfDNA of RA patients, which could partly explain the lower cfDNA methylation observed here. We did not explore the origin of the cfDNA in this research; however, measuring DNA methylation in cfDNA has the advantage of providing a systemic measure independent from cell-specific profiles.

The association between *DUSP22* DNA hypomethylation and increased joint space narrowing and SENS found in our study are consistent with previously published reports, which demonstrated that lower DNA methylation in the promoter region of *DUSP22* in monocytes, B-lymphocytes, naive CD4+ T and memory CD4+ T cells was associated with erosive disease (9). The CpG sites that showed an association here were also included in that study. However, the measures of erosive RA used in both studies were different. Mok and co-workers used the European League Against Rheumatism (EULAR) criteria, and here we used SENS, which is an indicator of erosive disease severity. However, the EULAR criteria describe the presence or absence of erosive disease. However, both studies agree that presence or severity of erosion in RA is associated with loss of DNA methylation at the *DUSP22* promoter region. This strengthens our findings’ validity as similar associations were observed in our study.

Our findings show that decreased DNA methylation of *DUSP22* at specific sites is associated with RA and joint space narrowing is connected to some of the known functions of *DUSP22*. The *DUSP22* protein activates JNK, which in turn promotes CD4+ T-cell differentiation (15). It acts as a scaffold protein that supports the formation of the Apoptosis Signal-regulating Kinase-1 (ASK1), Mitogen-activated protein Kinase (MAPK) Kinase-7 (MKK7), and JNK complex (16). In RA patients, the JNK pathway is characterized by heightened activity and has a broad role in different biological pathways relevant to the disease (reviewed in (35)). More recent studies have shown that JNK also promotes autophagy (36). Current research has linked this cellular process to bone erosion in mice and has found that RA patients have increased expression of autophagy-related proteins (37, 38). In addition, several studies have explored the connection between cfDNA’s presence and the underlying mechanisms of RA, particularly inflammation. Previous published research has shown that the presence of pro-inflammatory molecules promotes the loss of DNA methyltransferase expression in synoviocytes, which in turn results in lower DNA methylation in this tissue (33). Other work has shown that the presence of synovial cfDNA leads to increased inflammatory cytokine expression, and this increase has been attributed to the low DNA methylation levels in the cfDNA (34). Research has shown that these hypomethylated DNA sequences can act as ligands to the TLR-9 receptor, promoting inflammation, and affecting the clearance of cfDNA from plasma (30, 39). This earlier data points to more than one potential way in which lower cfDNA methylation and inflammation connect and highlight the interplay of several aspects of cfDNA processing that might impact considerations of this biomarker in future research. More research is also needed to understand the biological processes associated with DUSP22 and the pathogenesis of RA. Elucidating these mechanisms will help in the development of biomarkers that have the potential to improve response to treatment or the search for new therapies.

This study has some important limitations. The main one is its small number of participants, which may reduce statistical power to detect relevant associations. Taking this into consideration, we discussed correlations with *p*-values between 0.05 and 0.10 as trends and include effect sizes (η^2^) to provide context for the preliminary findings we present. We consider the age difference between RA and control participants a major limitation because DNA methylation changes with age, limiting the strength of the comparisons between RA cases and controls. Genome-wide methylation studies have not shown age-related changes in *DUSP22* among healthy individuals and we observed a similar pattern even within our smaller sample (40, 41). For RA patients, we had limited information on disease duration, so we could not examine whether the association between *DUSP22* methylation and age depended on length of disease. The association we observed between age and *DUSP22* DNA methylation in RA patients must be interpreted with caution and cannot be considered independent of cumulative disease burden. We also could not measure *DUSP22* protein levels, only DNA methylation in the promoter region. However, previous data measuring DNA methylation and gene expression suggests that *DUSP22* gene expression is regulated by DNA methylation of CpG sites in its promoter region (42). In addition, we did not compare measurements of DNA methylation in plasma cfDNA with those on other tissues relevant in RA. To address these limitations, future studies in a larger and independent cohort designed with rigorous age-matching and including disease duration information are urgently Needed to validate the exploratory findings presented here.

Our important strength of our pilot study is that it is the first one to investigate DNA methylation and RA associations in a predominantly Hispanic population. Hispanics are among the populations underrepresented in RA research and studies that explore potential biological mechanisms of the underlying disease in these groups are needed. Between 1995 and 2014, the incidence of RA remained stable for the overall population; but increased among Hispanic individuals (43). In a study comparing White, African American, Hispanic, and Asian RA patients found that Hispanics had higher disease activity and African Americans were less likely to reach clinical remission (44). These differences persisted after adjusting for socioeconomic and clinical factors, suggesting that disparities in RA are not driven solely by environmental influences (44). Ethnic variation in genetic susceptibility to RA has also been reported, indicating that biological factors may contribute to health disparities (45, 46). Studies focused on minority RA populations are scarce, and addressing this gap is essential to reduce inequities in disease outcomes.

In conclusion, we found that lower *DUSP22* DNA methylation in plasma cfDNA was associated with RA, and that these epigenetic differences were correlated with radiographic and disease activity measures. However, given our small sample size and the borderline statistical significance of our findings, we strongly suggest these results be considered hypothesis generating. Because cfDNA is easily obtainable, it may provide a practical source for systemic biomarker assessment and for monitoring treatment response. Our results highlight a possible role for cfDNA-based DNA methylation markers in RA, particularly in Hispanic patients, and report for the first time associations between *DUSP22* DNA methylation and radiographic features in this population. Future validation studies with a larger and multi-ethnic age-matched cohort and a prospective design can aid assess DNA methylation dynamics and treatment response and clinical characteristics of the disease. These future research should also explore the tissue of origin for the methylated cfDNA. Overall, this exploratory study expands our current knowledge on the epigenetic landscape of RA and supports further investigation of cfDNA methylation as a biomarker of disease severity and outcome.

## Data Availability

The raw data supporting the conclusions of this article will be made available by the authors, without undue reservation.
