# Supplementary material for: Dual Specificity Phosphatase (DUSP22) promoter hypomethylation in cell-free DNA is associated with rheumatoid arthritis and its radiographic severity
Source: Front Med (Lausanne). 2026 Feb 3;13:1730527. doi: 10.3389/fmed.2026.1730527 (PMC12909564; doi:10.3389/fmed.2026.1730527)
Supplement: Supplementary file 1 [file Data_Sheet_1.docx]

Supplementary Material

# Supplementary Tables

**Supplementary Table 1*.*** Analysis of Covariance (ANCOVA) of *DUSP22* DNA methylation percentages for RA patients and controls in the ERA study.

| **% DNA methylation** | **Mean†** | | **η²** | ***p*-value** |
| --- | --- | --- | --- | --- |
|  | **RA** | **Control** |  |  |
| ***CpG 1*** | *-0.88* | *-0.22* | *0.14* | ***0.049*** |
| ***CpG 2*** | *-0.74* | *-1.17* | *0.07* | *0.089* |
| ***CpG 3*** | *-0.69* | *-1.29* | *0.03* | *0.299* |
| ***CpG 4*** | *-1.14* | *-0.63* | *0.02* | *0.387* |
| ***Mean**** | *-0.88* | *-0.21* | *0.03* | *0.340* |

*****Mean % DNA methylation is an average of the measurements at each site, † logit-transformed DNA methylation percentages, η² are partial eta-squared values that provide effect size which can be interpreted according to Cohen’s thresholds, with 0.01 considered small, 0.06 medium, and 0.14 large. DNA methylation values were not normally distributed (Shapiro–Wilk *p*≤0.05 for all CpG site and the mean); however t the equality of variances assumption was satisfied for all sites (Levene’s test, *p*>0.05 for all CpG site and the mean). Given ANCOVA’s robustness under these conditions, the analyses remain valid and appropriately controlled for the covariate age.

**Supplementary Table 2.** Medication use and *DUSP22* DNA methylation percentages for RA patients and controls in the ERA study.

| ***Medication, N value*** | ***% DNA methylation*** | | | | |
| --- | --- | --- | --- | --- | --- |
|  | ***CpG 1*** | ***CpG 2*** | ***CpG 3*** | ***CpG 4*** | ***Mean**** |
| ***Methotrexate Use*** | | | | | |
| *Methotrexate, n=14* | *33.1 ± 4.3* | *35.5 ± 4.5* | *36.3 ± 4.9* | *29.5 ± 3.4* | *33.6 ± 4.2* |
| *No Methotrexate, n=12* | *40.3 ± 4.8* | *42.2 ± 4.8* | *44.6 ± 5.5* | *36.1 ± 3.9* | *40.8 ± 4.6* |
| *p-value* | *0.32* | *0.32* | *0.25* | *0.27* | *0.32* |
| ***TNFα Inhibitor Use †*** | | | | | |
| *TNFα Inhibitor, n=6* | *25.5 ± 5.3* | *28.3 ± 6.2* | *27.3 ± 7.2* | *23.8 ± 7.1* | *26.2 ± 6.3* |
| *No TNFα Inhibitor, n=20* | *39.7 ± 3.8* | *41.7 ± 3.8* | *44.0 ± 4.2* | *35.1 ± 2.6* | *40.1 ± 3.5* |
| *p-value* | *0.06* | *0.11* | *0.08* | *0.14* | *0.07* |

## Supplementary Figures


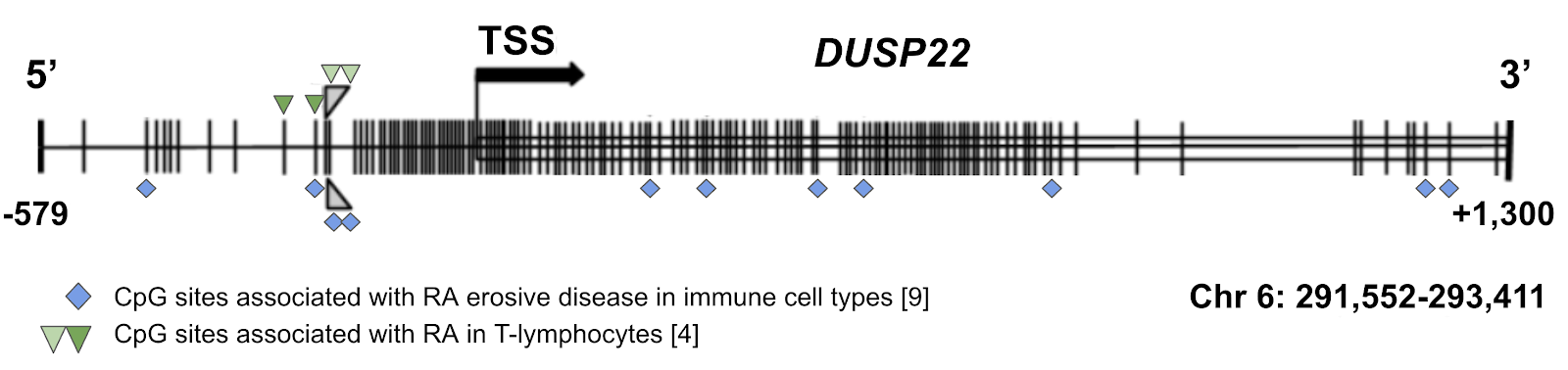


**Supplementary Figure 1.** **Map of the location of DNA methylation sites at the *DUSP22* promoter and around the transcription start-site (TSS).** A 1,860kb fragment in chromosome 6 that includes the first 1.3kb of the *DUSP22* gene and 580bp from the promoter region are shown. Across the sequence, lines are used to indicate the presence of CpG sites. DNA methylation sites included in previous studies are indicated in blue and green (4), (9). All CpG sites indicated with blue rhombi were measured using DNA methylation arrays (9). Light green triangles represent CpG sites uncovered through DNA methylation arrays, while dark green triangles represent CpG sites measured by pyrosequencing (4). All CpG sites marked in green were measured in this study. Genomic locations correspond to genome assembly 37.
